# Botulinum toxin therapy in the SARS-CoV-2 pandemic: patient perceptions from a German cohort

**DOI:** 10.1007/s00702-020-02235-6

**Published:** 2020-07-30

**Authors:** Dirk Dressler, Fereshte Adib Saberi

**Affiliations:** 1 Movement Disorders Section, Department of Neurology, Hannover Medical School, Carl-Neuberg-Str. 1, 30625 Hannover, Germany; 2 IAB-Interdisciplinary Working Group for Movement Disorders, Hamburg, Germany

**Keywords:** Botulinum toxin, Therapy, Corona virus, SARS-CoV-2, COVID19, Pandemic, Dystonia, Spasticity, Hemifacial spasms, Pain conditions, Quality of life, Perception, Patient rights

## Abstract

The SARS-CoV-2 virus pandemic has provoked drastic countermeasures including shutdowns of public services. We wanted to describe the effects of a 6 week shutdown of a large German botulinum toxin (BT) outpatient clinics on patients and their well-being. 45 patients (age 61.9 ± 9.8 years, 29 females, 16 males) receiving BT therapy (319.3 ± 201.9MU-equivalent, treatment duration 8.3 ± 5.5 years) were surveyed with a standardised questionnaire. The shutdown delayed BT therapy by 6.6 ± 2.3 weeks. 93% of the patients noticed increased muscle cramps and 82% increased pain reducing their quality of life by 40.2 ± 19.5%. For 23 patients with cervical dystonia this reduction was 41.1 ± 18.3%, for 3 patients with blepharospasm 33.3 ± 15.3%, for 9 patients with spasticity 37.8 ± 15.6%, for 4 patients with pain conditions 37.4 ± 35.7% and for 3 patients with hemifacial spasm 27.5 ± 17.1%. After the shutdown 66% of patients perceived BT therapy as more important than before, 32% perceived it as unchanged. For all patients long-term availability of BT therapy was very important or important. 98% of the patients perceived the shutdown as inadequate and felt their patient rights not respected. The shutdown confirmed the considerable burden of disease caused by dystonia, spasticity, hemifacial spasm and various pain conditions and the importance of BT therapy to treat them. Any shutdown severely affects these patients and needs to be avoided.

## Introduction

The corona crisis, the SARS-CoV-2 (corona) virus pandemic producing the clinical picture of COVID19, has hit virtually all health care systems worldwide with dramatic impact. Drastic corona virus countermeasures have been installed to deal with it. Many of them have deeply affected social and economic interactions, whilst others have affected the health care systems themselves. We wanted to describe how these countermeasures have affected patients receiving botulinum toxin (BT) therapy in Germany.

## Methods

### Design

The study is a survey of the effects of a 6 week BT outpatient clinics shutdown on its patients and their wellbeing. It is based on a face-to-face interview using the structuralised questionnaire shown in Table 1. Before presenting the questionnaire, patients were informed about the initiative of the ministry of health, its intention and its execution by Hannover Medical School and the Department of Neurology resulting in several countermeasures including a general shutdown of the BT outpatient clinics. The study was approved by the local ethics committee.

**Table 1:** Structuralised questionnaire to survey the effects of the anti-corona virus shutdown on patients receiving BT therapy

Symptoms caused by shutdown	Increased muscle cramps	O
	Increased muscle pain	O
Reduction of quality of life caused by shutdown (pls. estimate overall percentage)		□
Change of perception of BT therapy caused by shutdown	Perceive BT therapy as more important than before	O
	No change in perception	O
	Perceive BT therapy as less important than before	O
Perception of long-term BT treatment security	Very important	O
	Important	O
	Less important	O
Perception of the anti-corona shutdown	Inadequate	O
	Adequate	O
Respect of your patient rights	Not respected by shutdown	O
	Respected by shutdown	O

Translation from German

*BT* botulinum toxin

### Treatment institution

The study was performed at the Movement Disorders Section, Department of Neurology, Hannover Medical School, Hannover, Germany which was founded 11 years ago by one of the authors (DD) and is specialised in BT therapy. Currently, its annual BT usage is equivalent to more than 20,000 100MU vials of all major brands available in Germany.

### Patients

Patients were consecutively included in this study until the pre-set number of participants of 45 was reached. Patients had to fit the following inclusion criteria: (1) BT therapy delay due to the shutdown of more than 2 weeks. (2) BT therapy for more than 1 year. (3) Regular subjective global improvement induced by BT therapy > 30%. (4) No additional stress events after the last BT injection series. (5) Ability to understand and to react adequately to the questionnaire. The patient's participation in this survey was entirely voluntary. None of the patients invited declined their participation.

### BT therapy

BT therapy was performed with onabotulinumtoxinA (ONA, Botox®, Allergan GmbH, Frankfurt/M, Germany) (100MU in 2.5 ml 0.9% NaCl/H_2_O) incobotulinumtoxinA (INCO, Xeomin®, Merz Pharmaceuticals, Frankfurt/M Germany) (100MU in 2.5 ml 0.9% NaCl/H_2_O), or abobotulinumtoxinA (ABO, Dysport®, Ipsen Pharma GmbH, Munich, Germany) (500MU in 5.0 ml 0.9% NaCl/H_2_O). For comparison reasons equivalence mouse units (MU-E) were introduced. 1MU-E was equal 1MU of ONA and 1MU of INCO and 3MU of ABO. BT therapy is available to all patients at no costs except for a small nominal drug delivery charge. There is maximal freedom to determine interinjection intervals and BT doses.

### Corona virus countermeasures

Since early March 2020 the SARS-CoV-2 virus pandemic puzzled the public in Germany more and more. On March 17th 2020 the State of Lower Saxony Ministry of Health (Niedersächsisches Ministerium für Soziales, Gesundheit und Gleichstellung) ordered all hospitals […] to stop all un-initiated non-urgent medical procedures (Niedersächsische Verordnung zur Bekämpfung der Corona-Virus-Krankheit COVID-19) (Niedersächsisches Gesetz- und Verordnungsblatt 2020a). This was meant to release capacities for treatment of COVID patients and to reduce general infection risks. 6 weeks later, on May 5th this order was de-activated (Niedersächsisches Gesetz- und Verordnungsblatt 2020b). Referring to this order the Chairman of the Department of Neurology of Hannover Medical School closed the BT outpatient clinics (shutdown). Patients were informed that the clinics were closed for an unforeseeable period of time and that they should re-apply for an appointment again at a later point of time.

## Results

### Patients

As shown in Table 2, altogether 45 patients (age 61.9 ± 9.8 years, 29 females, 16 males) were included in this study. 23 patients suffered from cervical dystonia, 3 from blepharospasm, 9 from spasticity, 4 from pain conditions, 3 from hemifacial spasm.

**Table 2:** Patient demographics and botulinum toxin treatment data

Total number of patients [*n*]	45
Male patients [*n*]	16
Female patients [*n*]	29
Patient age (mean ± standard deviation) [years]	61.9 ± 9.8
Indication for BT therapy [*n*]	Cervical dystonia: 23
	Blepharospasm: 3
	Spasticity: 9
	Pain: 4
	Hemifacial spasm: 3
BT drug	AbobotulinumtoxinA: 3
	IncobotulinumtoxinA: 30
	OnabotulinumtoxinA: 12
BT total equivalent dose (mean ± standard deviation) [MU-E]	319.3 ± 201.9
Treatment duration (mean ± standard deviation) [years]	8.3 ± 5.5
Interinjection interval (mean ± standard deviation) [weeks]	11.2 ± 1.3
Subjective global improvement induced by BT therapy (mean ± standard deviation) [%]	67.2 ± 16.2

1MU-E = 1MU-onabotulinumtoxinA = 1MU-incobotulinumtoxinA = 0.5MU-abobotulinumtoxinA

*BT* botulinum toxin, *MU-E* equivalent mouse units

### BT therapy

As shown in Tables 2, 3 patients received ABO, 30 INCO and 12 ONA. Their BT total doses were 319.3 ± 201.9MU-E (min. 24MU-E, max. 900MU-E). They had been treated with BT therapy for 8.3 ± 5.5 years. Before the shutdown BT therapy was applied at intervals of 11.2 ± 1.3 weeks. The BT-induced improvement was 67.2 ± 16.2%.

**Table 3:** Effects of the botulinum toxin outpatient clinics shutdown on the patients and their well-being

Delay of re-treatment (mean ± standard deviation) [weeks]	6.6 ± 2.3
Symptoms caused by shutdown	
Increased muscle cramps [% of patients]	93
Increased muscle pain [% of patients]	82
Reduction of quality of life caused by shutdown	
All indications (*n* = 45) (mean ± standard deviation) [%]	40.2 ± 19.5
Cervical dystonia (*n* = 23) (mean ± standard deviation) [%]	41.1 ± 18.3
Blepharospasm (*n* = 3) (mean ± standard deviation) [%]	33.3 ± 15.3
Spasticity (*n* = 9) (mean ± standard deviation) [%]	37.8 ± 15.6
Pain conditions (*n* = 4) (mean ± standard deviation) [%]	37.4 ± 35.7
Hemifacial spasm (*n* = 4) (mean ± standard deviation) [%]	27.5 ± 17.1
Change of perception of BT therapy caused by shutdown	
BT therapy is more important than before [% of patients]	66
No change [% of patients]	32
BT therapy is less important than before [% of patients]	2
Perception of long-term BT treatment security	
Very important [% of patients]	91
Important [% of patients]	9
Less important [% of patients]	0
Perception of shutdown	
Inadequate [% of patients]	98
Adequate [% of patients]	2
Respect of your patient’s rights	
Not respected [% of patients]	98
Respected [% of patients]	2

### Shutdown effects

Table 3 shows the effects of the shutdown on the patients and their well-being. As the result of the shutdown, the interinjection intervals increased from 11.2 ± 1.3 weeks to 17.7 ± 2.4 weeks delaying BT therapy by 6.6 ± 2.3 weeks. The minimum delay was (by definition) 2 weeks, the maximum delay 12 weeks. 93% of the patients noticed increased muscle cramps and 82% increased muscle pain. Due to the shutdown, the patient's quality of life was reduced by 40.2 ± 19.5%. For patients with cervical dystonia this reduction was 41.1 ± 18.3%, for patients with blepharospasm 33.3 ± 15.3%, for patients with spasticity 37.8 ± 15.6%, for patients with pain condition 37.4 ± 35.7% and for patients with hemifacial spasm 27.5 ± 17.1%. After the shutdown 66% of patients perceived BT therapy as more important than before. For 32% of patients the perception of BT therapy was unchanged. One patient (2%) considered BT therapy as less important after the shutdown, perceived the shutdown as adequate and felt her patient rights adequately respected. 91% of patients perceived long-term security of BT therapy availability as very important, 9% as important and none as less important. 98% of the patients perceived the shutdown as inadequate and felt that their patient rights were not respected.

## Discussion

The corona crisis has provoked numerous countermeasures. One of the most invasive ones are shutdowns, especially when they affect medical services. There have been several occasions when medical services were shutdown in the past. They included trade union actions, natural disasters, computer virus attacks and war activities. Never before, however, have medical service shutdowns lasted so long and have they affected such a variety of services and such large geographical areas. This massive impact should have produced signals strong enough for various analyses including the relevance of the treated disorders and the BT therapy offered.

In our study the shutdown increased the interinjection intervals from 11.2 ± 1.3 to 17.7 ± 2.4 weeks delaying BT therapy by 6.6 ± 2.3 weeks. In 38% of patients the delay was more than the actual duration of the shutdown as re-scheduling of these patients was problematic with the reduced capacity of the re-opened BT outpatient clinics. With BT’s natural time course of action (Dressler and Adib Saberi 2005) this delay will have washed out most of BT’s biological activity. Subsequently, the patient's quality of life during this period was reduced by 40.2 ± 19.5%. There were no apparent differences between the different indications. This means that even indications such as hemifacial spasm which may be perceived as less severe may produce a reduction of quality of life similar to indications which may have been perceived as more sever such as cervical dystonia or spasticity. More specifically and depending on the treated indication muscle hyperactivity and muscle pain have re-occurred during the shutdown in almost all patients.

Sixty-six percent of patients perceived their BT therapy as more important than before the shutdown experience. In 32% of patients the shutdown experience confirmed the patients' perception of the importance of their BT therapy. One patient (2%) reported a lack of worsening of her cervical dystonia. She felt she may not need their BT therapy any more. Careful re-evaluation indicated that her mild cervical dystonia had recently improved and that the indication for her treatment has become relative, thus underlining the importance of occasional re-evaluations of patients receiving chronic BT therapy.

All patients expressed that the security of long-term availability of their BT therapy was very important or important. Contrary to the patient's assumptions and expectations this security was obviously not provided by a large tertiary referral centre. Several patients expressed their disappointment about this fact.

Ninety-eight percent of patients felt their patient rights not respected. It is irritating that decision makers are not perceiving dystonia, spasticity and certain pain conditions as not severe enough to trigger treatment. Respective patient groups may use this information to intensify their advocacy and public awareness efforts in the future.

The shutdown confirmed the considerable burden of disease caused by dystonia, spasticity, hemifacial spasm and various pain conditions and the importance of BT therapy to treat them. Interrupting this therapy for whatever reason may generate substantial reductions in their well-being. Any shutdown of BT therapy may severely affect patients and needs to be avoided.
